# Supplementary material for: Dissociable roles of the inferior longitudinal fasciculus and fornix in face and place perception
Source: eLife. 2015 Aug 29;4:e07902. doi: 10.7554/eLife.07902 (PMC4586481; doi:10.7554/eLife.07902)
Supplement: Supplementary file 1. — Table of co-ordinates for the whole-brain tract-based spatial statistics (TBSS) analysis. DOI: http://dx.doi.org/10.7554/eLife.07902.016 [file elife07902s004.docx]

**Supplementary File 1.** Table of co-ordinates for the whole brain TBSS analysis.

| **Metric** | **Cluster** | **Voxels** | **P** | **x** | **y** | **z** | **Peak voxel location** |
| --- | --- | --- | --- | --- | --- | --- | --- |
| **MD** | 1 | 9473 | 0.018 | 31 | -33 | 37 | SLF |
|  | 2 | 455 | 0.043 | -19 | -50 | 21 | Splenium of corpus callosum |
|  | 3 | 388 | 0.047 | -17 | -54 | 51 | Superior parietal white matter |
|  | 4 | 222 | 0.047 | 27 | 7 | 24 | Superior corona radiata |
|  | 5 | 180 | 0.045 | -14 | -31 | 55 | Corticospinal tract |
|  | 6 | 102 | 0.048 | -25 | -41 | 44 | SLF |
|  | 7 | 75 | 0.049 | 27 | -6 | 32 | Corticospinal tract |
|  | 8 | 73 | 0.047 | -10 | -29 | 34 | Cingulum |
|  | 9 | 52 | 0.05 | 48 | -16 | 40 | Postcentral gyrus white matter |
|  | 10 | 49 | 0.05 | 23 | 2 | 35 | Superior corona radiata |
|  | 11 | 47 | 0.049 | 19 | -16 | -8 | Corticospinal tract |
|  | 12 | 27 | 0.05 | -14 | -34 | 35 | Cingulum |
|  | 13 | 22 | 0.05 | 40 | -25 | 48 | Postcentral gyrus white matter |
|  | 14 | 12 | 0.05 | 7 | -56 | 13 | Cingulum |
|  | 15 | 10 | 0.05 | -7 | -34 | 21 | Splenium of corpus callosum |
|  | 16 | 10 | 0.05 | 42 | -29 | -11 | ILF |
|  | 17 | 7 | 0.05 | 12 | -18 | 62 | Precentral gyrus white matter |
|  | 18 | 7 | 0.05 | 29 | -52 | 17 | Corpus callosum |
|  | 19 | 3 | 0.05 | 18 | -54 | 33 | Corpus callosum |
|  | 20 | 2 | 0.05 | 16 | -39 | 61 | Corticospinal tract |
|  | 21 | 2 | 0.05 | 52 | -13 | 40 | Postcentral gyrus white matter |
|  | 22 | 1 | 0.05 | 17 | -54 | 61 | Superior parietal white matter |
|  | 23 | 1 | 0.05 | 9 | -58 | 57 | Precuneous white matter |
| **FA** | 1 | 31383 | 1 | 16 | 12 | 29 | Corpus callosum |
|  | 2 | 118 | 1 | 40 | -33 | -15 | ILF |
|  | 3 | 101 | 1 | -8 | -63 | 25 | Cingulum |
|  | 4 | 83 | 1 | -49 | -35 | -14 | SLF |
|  | 5 | 45 | 1 | 16 | 57 | -3 | Forceps minor |
|  | 6 | 19 | 1 | 40 | -77 | 3 | ILF |
|  | 7 | 6 | 1 | 38 | -70 | -12 | Occipital fusiform white matter |
|  | 8 | 5 | 1 | 27 | 39 | -2 | IFOF |
|  | 9 | 1 | 1 | 55 | -2 | 16 | SLF |

**Supplementary File 1.** Table of co-ordinates for the whole brain Tract-Based Spatial Statistics (TBSS) analysis. Cluster peaks are reported for mean diffusivity (MD, top) and fractional anisotropy (FA, bottom). Abbreviated white matter structures include SLF (superior longitudinal fasciculus), IFOF (inferior fronto-occipital fasciculus) and ILF (inferior longitudinal fasciculus).
