# Supplementary material for: Dissociable roles of the inferior longitudinal fasciculus and fornix in face and place perception
Source: eLife. 2015 Aug 29;4:e07902. doi: 10.7554/eLife.07902 (PMC4586481; doi:10.7554/eLife.07902)
Supplement: Supplementary file 2. — Results of the mediation analyses across the three ROIs. DOI: http://dx.doi.org/10.7554/eLife.07902.017 [file elife07902s005.docx]

**Supplementary File 2.** Results of the mediation analyses across the three ROIs.

|  |  | **(a) ROIs** | | | **(b) ROIs by hemisphere** | | | |
| --- | --- | --- | --- | --- | --- | --- | --- | --- |
|  |  | ***FFA*** | ***PrC*** | ***HC*** | ***Left PrC*** | ***Right PrC*** | ***Left FFA*** | ***Right FFA*** |
| **Model 1** | ***a (p)*** | -1.99 (*p* = 0.00) | -1.1 (*p* = 0.24) | -3.62 (*p* = 0.31) | -0.68 (*p* = 0.34) | -0.74 (*p* = 0.31) | -1.99 (*p* = 0.02) | -1.65 (*p* = 0.00) |
|  | ***b (p)*** | -1852.78 (*p* = 0.05) | -1826.53 (*p* = 0.01) | -638.08 (*p* = 0.01) | -1155.74 (*p* = 0.05) | -2039.51 (*p* = 0.01) | -1317.98 (*p* = 0.01) | -1742.41 (*p* = 0.04) |
|  | ***a*b (CI)*** | 0.04 (0.006, 0.099) | 0.02 (-0.01, 0.10) | -0.02 (-0.001, 0.06) | 0.03 (-0.03, 0.07) | 0.02 (-0.05, 0.08) | 0.03 (-0.001, 0.07) | 0.03 (0.001, 0.09) |
|  | ***c' (p)*** | 0.01 (*p* = 0.39) | 0.11 (*p* = 0.03) | -0.15 (*p* = 0.03) | 0.07 (*p* = 0.08) | 0.11 (*p* = 0.02) | -0.01 (*p* = 0.42) | 0.02 (*p* = 0.21) |
|  | ***c (p)*** | 0.05 (*p* = 0.05) | 0.13 (*p* = 0.02) | -0.13 (*p* = 0.08) | 0.07 (*p* = 0.07) | 0.13 (*p* = 0.02) | 0.02 (*p* = 0.27) | 0.05 (*p* = 0.02) |
| **Model 2** | ***a (p)*** | -20869.18 (*p* = 0.00) | -2116.3 (*p* = 0.48) | -338.97 (*p* = 0.31) | -1218.63 (*p* = 0.34) | 1568.28 (*p* = 0.31) | -11281.1 (*p* = 0.1) | -21896.55 (*p* = 0.00) |
|  | ***b (p)*** | 0.01 (*p* = 0.39) | 0.11 (*p* = 0.06) | -0.15 (*p* = 0.03) | 0.07 (*p* = 0.08) | 0.11 (*p* = 0.02) | -0.01 (*p* = 0.42) | 0.02 (*p* = 0.21) |
|  | ***a*b (CI)*** | -200.37 (-1105.28, 1321.92) | -226.61 (-1232.97, 142.08) | 51.88 (-77.15, 590.53) | -87.07 (-1063.53, 260.43) | -178.84 (-913.02, 647.68) | 75.17 (-520.59, 1298.82) | -475.95 (-1522.49, 687.33) |
|  | ***c' (p)*** | -1852.78 (*p* = 0.05) | -1826.53 (*p* = 0.01) | -638.08 (*p* = 0.01) | -1155.74 (*p* = 0.05) | -2039.51 (*p* = 0.01) | -1317.98 (*p* = 0.06) | -1742.41 (*p* = 0.04) |
|  | ***c (p)*** | -2053.15 (*p* = 0.01) | -2053.15 (*p* = 0.01) | -586.2 (*p* = 0.02) | -1242.81 (*p* = 0.04) | -2218.35 (*p* = 0.01) | -1242.81 (*p* = 0.04) | -2218.35 (*p* = 0.01) |

**Supplementary File 4.** Results of the mediation analyses across our three *a priori* ROIs. In model 1 (top), blood oxygen level-dependent (BOLD) response is the independent variable, white matter microstructure is the mediator and oddity performance is the dependent variable. In model 2 (bottom), white matter microstructure is the independent variable and BOLD response is the mediator. Data are reported for the fusiform face area (FFA, left), perirhinal cortex (PrC, middle) and hippocampus (HC, right). **(b)** Results of the mediation analysis when comparing left and right hemispheres for the PrC (top) and FFA (bottom). In model 1, BOLD response is the independent variable (left vs. right), inferior longitudinal fasciculus (ILF) microstructure for the respective hemisphere is the mediator, and face oddity performance is the dependent variable. In model 2, ILF microstructure (left vs. right) is the independent variable and BOLD response for the respective hemisphere is the mediator. Unstandardised coefficients and one-tailed *p*-values are reported for all models paths (*a, b, a*b, c', c*). Coefficients relating to changes in MD are formatted *n*(x 10^-5^mm^2^/s). The 95% confidence interval (CI) is reported for the indirect effect (*a*b*) and is based on 1000 bootstrapped iterations.
